# Genotype-relevant neuroimaging features in low-grade epilepsy-associated tumors

**DOI:** 10.3389/fneur.2024.1419104

**Published:** 2024-07-16

**Authors:** Keiya Iijima, Hiroyuki Fujii, Fumio Suzuki, Kumiko Murayama, Yu-ichi Goto, Yuko Saito, Terunori Sano, Hiroyoshi Suzuki, Hajime Miyata, Yukio Kimura, Takuma Nakashima, Hiromichi Suzuki, Masaki Iwasaki, Noriko Sato

**Affiliations:** ^1^Department of Neurosurgery, National Center Hospital, National Center of Neurology and Psychiatry, Kodaira, Tokyo, Japan; ^2^Department of Radiology, National Center Hospital, National Center of Neurology and Psychiatry, Kodaira, Tokyo, Japan; ^3^Medical Genome Center, National Center of Neurology and Psychiatry, Kodaira, Tokyo, Japan; ^4^Department of Pathology and Laboratory Medicine, National Center Hospital, National Center of Neurology and Psychiatry, Kodaira, Tokyo, Japan; ^5^Department of Neurology, Tokyo Metropolitan Geriatric Hospital and Institute of Gerontology, Tokyo, Japan; ^6^Department of Pathology and Laboratory Medicine, National Hospital Organization Sendai Medical Center, Sendai, Miyagi, Japan; ^7^Department of Neuropathology, Research Institute for Brain and Blood Vessels, Akita Cerebrospinal and Cardiovascular Center, Akita, Japan; ^8^Division of Brain Tumor Translational Research, National Cancer Center Research Institute, Tokyo, Japan

**Keywords:** low-grade epilepsy-associated tumors, neuroimaging features, genetic alterations, genotype, histopathology, Low-grade neuroepithelial tumor, LEAT

## Abstract

**Introduction:**

Low-grade epilepsy-associated tumors are the second most common histopathological diagnoses in cases of drug-resistant focal epilepsy. However, the connection between neuroimaging features and genetic alterations in these tumors is unclear, prompting an investigation into genotype-relevant neuroimaging characteristics.

**Methods:**

This study retrospectively analyzed neuroimaging and surgical specimens from 46 epilepsy patients with low-grade epilepsy-associated neuroepithelial tumors that had genetic mutations identified through panel sequencing to investigate their relationship to genotypes.

**Results:**

Three distinct neuroimaging groups were established: Group 1 had indistinct borders and iso T1-weighted and slightly high or high T2-weighted signal intensities without a diffuse mass effect, associated with 93.8% sensitivity and 100% specificity to *BRAF* V600E mutations; Group 2 exhibited sharp borders and very or slightly low T1-weighted and very high T2-weighted signal intensities with a diffuse mass effect and 100% sensitivity and specificity for *FGFR1* mutations; and Group 3 displayed various characteristics. Histopathological diagnoses including diffuse low-grade glioma and ganglioglioma showed no clear association with genotypes. Notably, postoperative seizure-free rates were higher in Group 1 tumors (*BRAF* V600E) than in Group 2 tumors (*FGFR1*).

**Discussion:**

These findings suggest that tumor genotype may be predicted by neuroimaging before surgery, providing insights for personalized treatment approaches.

## 1 Introduction

Low-grade epilepsy-associated tumors (LEAT) are the second most common histopathological diagnosis after cortical malformations in pediatric patients undergoing epilepsy surgery; they are found in 27.2% of surgical specimens (1, 2). LEAT include low-grade gliomas and glioneuronal tumors, collectively designated as “low-grade neuroepithelial tumors”; they are an important group of central nervous system neoplasms in children and young adults with epilepsy (3). Ganglioglioma (GG) and dysembryoplastic neuroepithelial tumor (DNT) are the most frequent pathological diagnoses among LEAT patients (4), accounting for >80% of tumors classified as LEAT and 65% of all brain tumors in a previous, large epilepsy surgery series (1). The term “LEAT” is one clinicopathological concept that has been the subject of research, but its definition is not well established.

Recent molecular genetic studies have revealed various mutations in the mitogen-activated protein kinase (MAPK) pathway, including *BRAF* and *FGFR1*, in low-grade neuroepithelial tumors (4–6); however, associations between genotypes and pathological findings remain unclear (5, 7, 8). The *BRAF* V600E mutation has been reported in GG, polymorphous low-grade neuroepithelial tumor of the young (PLNTY), pleomorphic xanthoastrocytoma, pilocytic astrocytoma, DNT, and MAPK pathway-altered diffuse low-grade glioma (dLGG). *FGFR1* mutations have been reported in DNT, PA, and MAPK pathway-altered dLGG (9–11). In these studies, surgical specimens were collected from surgeries for brain tumors; a part of samples were obtained from patients with no history of epilepsy. Therefore, genetic characteristics of the tumors that truly cause drug-resistant epilepsy are not sufficiently clear. However, the inconsistent associations between genotype and pathology appear to be attributed, in part, to the difficulty with histological interpretations of low-grade neuroepithelial tumors (12) because of sampling errors in surgical specimens for histopathological evaluation. However, several imaging modalities can capture the overall tissue characteristics, thus reducing the likelihood of misinterpretation. A recent study reported an association between MRI and pathological findings in LEAT (6), but no association between neuroimaging findings and genotypes.

Therefore, we hypothesized that genetic mutations are more likely to be associated with imaging findings than with pathological findings. We investigated the genotype-relevant neuroimaging features and their clinicopathological significance in patients with LEAT.

## 2 Materials and methods

**Figure 1:**
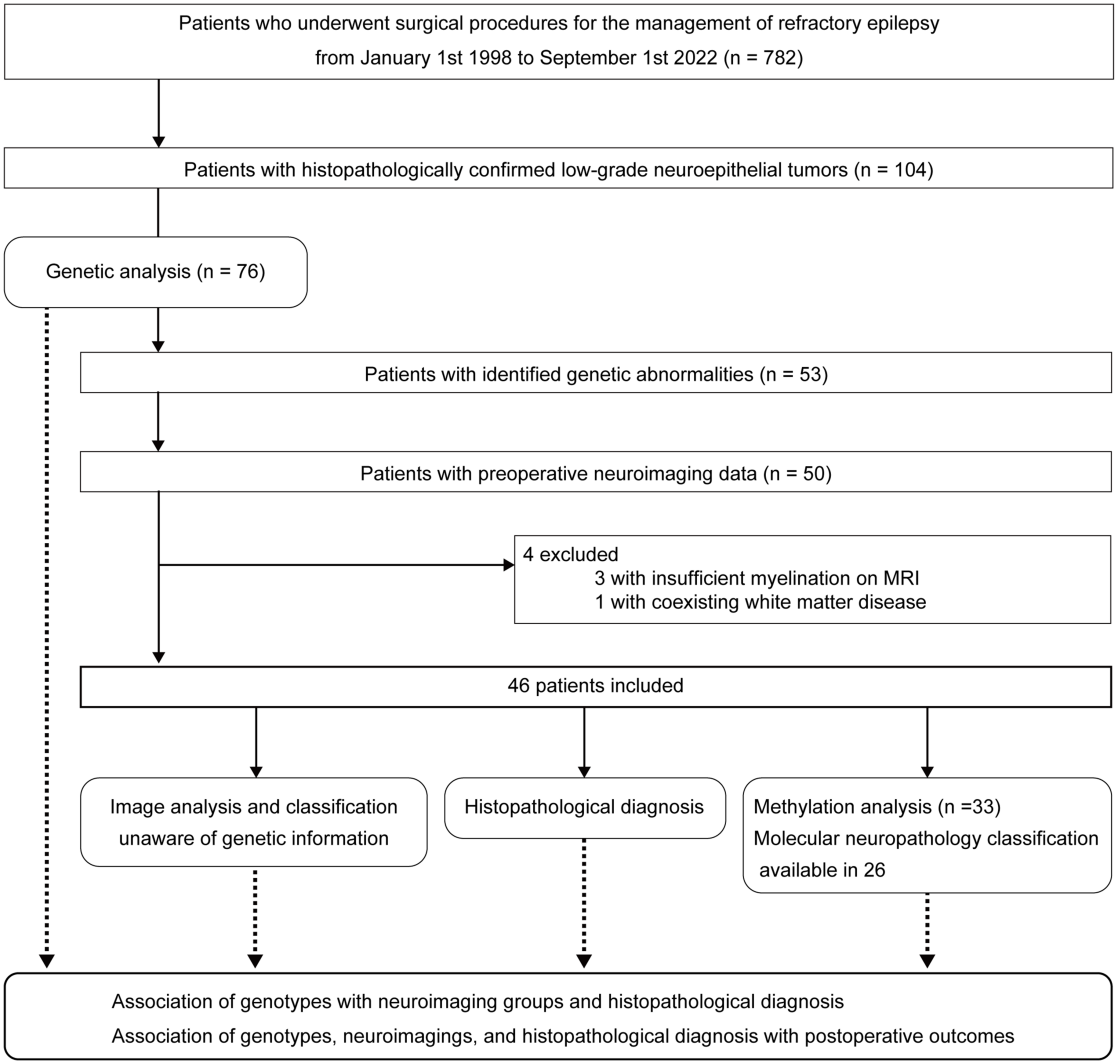
Patient selection and study outline.

This study adheres to the STROBE reporting guidelines. Patient selection and study outline are summarized in Figure 1.

### 2.1 Patients

This study included the following patients who had undergone surgical procedures at National Center Hospital, National Center of Neurology and Psychiatry, Tokyo, Japan.

Inclusion criteria

1. Patients who had undergone surgical procedure for drug-resistant focal epilepsy.
2. The pathological diagnoses at surgery were low-grade neuroepithelial tumors.
3. Patients who revealed with a known genetic mutation.
4. Patients for whom preoperative neuroimaging data were available.

Exclusion criteria

1. Difficulty in image evaluation due to insufficient myelination on MRI
2. Coexisting diffuse white matter disease

A retrospective survey of the neuropathology database at our institution identified 782 patients with drug-resistant epilepsy who had undergone surgical procedures for epileptogenic lesions between January 1, 1998, and September 1, 2022. We found 104 patients with histopathologically confirmed, low-grade neuroepithelial tumors and obtained informed consent from 79 patients. Three of the 79 patients were excluded because of insufficient tumor tissue available for this study. Genetic analyses of the surgical specimens were performed in 76 patients through panel sequencing targeting a set of LEAT-related genes. Twenty-three patients with gene panel-negative tumors, three without complete data for preoperative MRI, three with insufficient myelination on MRI, and one with coexisting diffuse white matter disease on MRI were further excluded from neuroimaging analysis. The three patients who were excluded because of insufficient myelination were aged 4, 5, and 7 months. In every case, we have checked the myelination as well as tumor imaging characteristics. When the signal of brain parenchyma around the tumor had already been myelinated, we thought it would be possible to accurately judge the tumor signals and the patient was included in the study. Consequently, 46 patients were finally included.

### 2.2 Clinical features

The following clinical information was retrieved from medical records: age at onset of epilepsy, age at surgery, surgical procedure, recurrence or regrowth of tumor, and postsurgical seizure outcomes evaluated 2 and 5 years after surgical intervention. Postoperative seizure outcome was classified according to the International League Against Epilepsy outcome scale (13). One case was excluded from the outcome evaluation because only a biopsy was performed for diagnostic purposes.

### 2.3 Histopathological diagnosis

Three neuropathologists (Y.S., H.S., and H.M.) examined the surgical specimens under a multi-head microscope to discuss the consensus histopathological and immunohistochemical features. Integrated diagnoses were made based on the 2021 WHO Classification of Tumors of the Central Nervous System (9), incorporating the molecular genetic results.

### 2.4 Genetic analyses

DNA preparation and extraction are described in Supplementary methods.

#### 2.4.1 Multiplex ligation-dependent probe amplification analysis

Principal genetic mutation patterns were identified using The multiplex ligation-dependent probe amplification (MLPA) technique with P088 and P370 MLPA kits (MRC Holland, Amsterdam, The Netherlands). The P370 Kit includes 58 probes for gene detection, including *BRAF*, *KIAA1549*, *IDH1/2*, *TACC1*, *CDKN2A/B*, and *FGFR1*. The P088 Kit includes 58 probes for gene detection, including *IDH1/2*, *CDKN2A/B*, *FGFR1*, and chromosomes 1 and 19. MLPA analyses were performed following The manufacturer’s instructions. The results were analyzed using The Coffalyser.net software (MRC Holland).

#### 2.4.2 Next-generation sequencing

Multiple primer sets covering exonic and exon–intron border regions (+25 to −25) of genes involved in LEAT (*IDH1*, *IDH2*, *BRAF*, *FGFR1*, *TP53*, *SLC44A1*, *PRKCA*, and *KIAA1549*) were designed using Ion AmpliSeq Designer software (Thermo Fisher Scientific). The coverage rates of the targets were 90%. Before the target amplification reaction, deaminated cytosine (uracil) bases were removed from the FFPE tissue by treatment with uracil DNA glycosylase (Invitrogen, Carlsbad, CA, United States). DNA derived from frozen or FFPE tissue (20 ng) was amplified using polymerase chain reaction with a premixed AmpliSeq HD Library Kit (Thermo Fisher Scientific) following the manufacturer’s protocol.

Each library containing 50 pM was loaded on an Ion Chef™ Instrument (Ion Torrent^™^; Thermo Fisher Scientific). Prepared libraries were loaded onto Ion 540 Chips (four samples/chip) and sequenced using an Ion GeneStudio S5 System (Thermo Fisher Scientific), aiming for a read length of 200 bp and 500 flow cycles.

#### 2.4.3 Sanger sequencing

Mutations with a frequency of >0.5% in next-generation sequencing were confirmed with Sanger sequencing on an automated DNA analyzer/sequencer (Applied Biosystems 3730xl; Thermo Fisher Scientific).

#### 2.4.4 DNA methylation profiling and analysis

Genome-wide DNA methylation profiling was performed for 33 patients using the Illumina Infinium Methylation EPIC BeadChip array (Illumina, San Diego, CA, United States). There were not enough DNA samples left for the methylation analysis in the remaining 13 patients. Classification of tumors was performed with the Molecular Neuropathology (MNP) classifier (14),^1^ using its newest version 12.8. The classifier classifies samples into a class. A calibrated classification score ≥ 0.9 (score ranging between 0 and 1) was considered a successful classification according to the instructions of the classifier. Samples with a calibrated score < 0.9 have been denoted here as “no match.”

### 2.5 Image analysis

#### 2.5.1 Image acquisition

Preoperative MR images were acquired with a 1.5-T or 3-T MR system (Magnetom Symphony or Magnetom Verio; Siemens, Erlangen, Germany; and Achieva, Philips Medical Systems, Best, The Netherlands) from 1 to 203 days (median 13 days) before surgery. T1-weighted, T2-weighted, and fluid-attenuated inversion recovery (FLAIR) images were obtained for all 46 patients. Diffusion-weighted, gadolinium-enhanced T1-weighted, and double inversion recovery (DIR) imaging were performed in 29, 27, and 20 patients, respectively. Preoperative CT was performed for 45 patients within 6 months before surgery. Details of image acquisition, including PET and SPECT image acquisitions, are provided in the Supplementary material.

#### 2.5.2 Image feature extraction

Three neuroradiologists (F.S., H.F., and N.S., with 9, 9, and 30 years of experience in neuroradiology, respectively) independently evaluated all images, unaware of the genetic information. Any differences in evaluations were resolved by consensus. MRI findings of the tumors were qualitatively evaluated, focusing on the following characteristics: location, size, border, shape, signal intensity, and the presence or absence of exophytic growth, mass effect, gadolinium enhancement, and cystic component, as well as coexisting hippocampal sclerosis. Tumor locations were classified into left or right frontal, temporal, parietal, and occipital lobes. The temporal lobe was further divided into medial and lateral temporal lobes with or without temporal base involvement by the tumor lesion. Tumor size was measured as the maximum length and the orthogonal width and height. Tumor shapes were classified as round, multilocular, triangular, rectangular, wedge, or band. Tumor borders were categorized as sharp or indistinct. We defined it as mass effect if the tumor was swollen toward the surrounding normal parenchyma. If the entire lesion was swollen, we defined it “diffuse mass effect,” and if a part of the lesion was swollen, we defined it “partial mass effect.” We defined it as exophytic growth if the tumor grows beyond the surface of the brain, occasionally accompanied with bony scalloping.

Signal intensity was evaluated on T1- and T2-weighted images and the ADC value was derived from the diffusion-weighted images. Heterogeneous or mixed-signal intensities were classified by evaluating the most representative area of the lesion. Signal intensity on T1-weighted images was classified into four categories based on visual findings: (1) high, showing iso- or higher intensity than the white matter; (2) iso, iso- or higher intensity compared with the cortex and lower than the white matter; (3) slightly low, lower intensity than the cortex and higher than the CSF; and (4) very low, iso-intense to the CSF. Signal intensity on T2-weighted images was classified into five categories based on visual findings: (1) very high, showing iso-intensity compared with the CSF; (2) high, higher intensity than the cortex and lower than the CSF; (3) slightly high, iso-intense compared to the cortex; (4) iso, iso- or higher intensity than the white matter and lower than the cortex; and (5) low, lower intensity than the white matter. The ADC values of the tumor were classified into four categories based on visual findings using an ADC map as follows: (1) very high, showing iso-intensity compared to the CSF; (2) slightly high, higher intensity than the surrounding brain tissue; (3) iso, iso-intense compared to the surrounding brain tissue; and (4) low, lower intensity than the surrounding brain tissue. Gadolinium enhancement was visually classified into three categories: (1) no enhancement, (2) faint or partial enhancement, and (3) homogeneous enhancement. The presence of cystic components was determined when the lesion showed iso-intensity compared to the CSF on T1-weighted, T2-weighted, and FLAIR images. The tension of the cystic components was classified as tense or flaccid. Hippocampal sclerosis was defined by reduced hippocampal volume with increased signal intensity on T2-weighted images. The presence or absence of skull scalloping and calcification was evaluated on CT. Tracer uptake on ^18^FDG-PET and ethyl-cysteinate-dimer-SPECT was categorized into increased, equivalent, or decreased compared with the contralateral normal brain tissues.

The inter-rater agreement was evaluated to determine the reliability of the image feature extraction. We calculated Fleiss’ Kappa coefficient for categorical variables (border, shape, exophytic growth, mass effect, cystic component, coexisting hippocampal sclerosis, skull scalloping, calcification, FDG-PET, SPECT) and the intraclass correlation coefficient for ordinal variables (signal intensities of T1- and T2-weighted images and the ADC values and gadolinium enhancement).

#### 2.5.3 Hierarchical clustering analysis

Hierarchical cluster analysis was performed to identify groups of patients with similar neuroimaging characteristics. Clustering of neuroimaging features was performed based on squared Euclidean distance with Ward’s algorithm using IBM SPSS Statistics version 27 or higher (IBM Corp., Armonk, NY, United States). Four binary variables (tumor border, exophytic growth, cystic component, and calcification) and three ordinal variables (mass effect and T1 and T2 signal intensities) were included in the analysis.

### 2.6 Associations between genotypes, neuroimaging groups, histopathological diagnosis, and postsurgical outcome

The association of genotypes with the identified imaging groups and histopathological diagnosis was examined. The sensitivity and specificity of the neuroimaging groups and pathological diagnoses for the detection of genotypes were calculated. The Fisher’s exact test with the Holm–Bonferroni correction was performed to analyze the association of preoperative characteristics (genotype, neuroimaging group, and histopathological diagnosis) with postoperative outcomes. Statistical analyses were performed with R version 4.0.2 (The R Foundation for Statistical Computing, Vienna, Austria). Statistical significance was considered when the *p-*value was <0.05.

### 2.7 Standard protocol approvals, registrations, and patient consent

This was a retrospective descriptive study. Clinical information and specimens were obtained with written informed consent by participants and/or their legal guardians. This study was approved by the National Center of Neurology and Psychiatry Ethics Committee, Japan (NCNP-A2018-050) and performed in accordance with the Declaration of Helsinki. All methods were performed in accordance with the relevant guidelines and regulations.

## 3 Results

### 3.1 Clinical features, genotypes, molecular neuropathology classes, and histopathological diagnosis

The study included 46 patients comprising 25 males and 21 females aged 7.2 ± 5.9 years (mean ± standard deviation; range, 0–18 years) at epilepsy onset, and their age was 13.8 ± 9.8 years (range, 1–38 years) at the first surgery. The epilepsy duration was 6.6 ± 7.2 years (range, 0–32 years). None of the patients had apparent skeletal or skull deformities. The complete seizure-free rates 2 and 5 years after the first surgery were 88.9% (40/45) and 85.7% (24/28), respectively. A second surgery for recurrent tumors was performed 2–7 years after the first surgery in five patients (Patients 8, 21, 27, 28, and 36), and the residual tumor was confirmed histopathologically in all five patients (Table 1). Four of these five patients also had recurrent seizures. Seizure freedom was achieved in three of the four patients 2 years after the second surgery. No patients underwent a third surgery. Complete seizure freedom was achieved in 75.6% (34/45) of the patients at the last follow-up examination. All three patients with hippocampal sclerosis underwent anterior temporal lobectomy with amygdalo-hippocampectomy and showed freedom from seizures at 2 years after surgery.

**Table 1:** Clinical characteristics and postoperative outcome in 46 patients with LEAT.

Patient number	Sex	Age at onset (years)	Age at surgery (years)	Epilepsy duration (years)	Surgical procedure	Tumor outcome		Seizure outcome^a^			
						Tumor remnant	Recurrence / regrowth	2 years	5 years	Last follow-up	Follow-up duration (months)
1	M	15	19	4	Lesionectomy	−	−	1a	1a	1a	155
2	M	2	11	9	Lesionectomy	−	−	1a	1a	1a	137
3	M	15	27	12	Lesionectomy	−	−	1a	1a	1a	211
4	M	15	30	15	ATL	−	−	1a	1a	1a	170
5	M	10	34	24	ATL with H-tomy	−	−	1a		1a	27
6	F	14	15	1	ATL with H-tomy	−	−	1a	1a	4	188
7	M	0	1	1	ATL	+	−	1a	1a	1a	184
8	M	12	13	1	ATL	−	+	1a	1a	1*	183
9	F	12	13	1	Lesionectomy	−	−	1a		1a	35
10	M	0	9	9	ATL with H-tomy	−	−	1a	1a	1a	69
11	M	13	22	9	Lesionectomy	−	−	1a	1a	1a	172
12	M	2	3	1	Lesionectomy	+	+	1a	1a	1a	142
13	M	1	3	2	Biopsy	+	+				166
14	M	1	4	3	ATL with H-tomy	−	−	1a	1a	1a	88
15	M	0	10	10	ATL with H-tomy	−	−	1a	1a	1a	78
16	F	18	23	5	ATL with H-tomy	−	−	1a	1a	1a	58
17	F	16	25	9	ATL with H-tomy	−	−	1a	1a	1a	143
18	M	10	10	0	ATL with H-tomy	−	−	1a	1a	1a	151
19	M	7	11	4	Lesionectomy	−	−	1a	1a	1a	144
20	M	15	29	14	ATL	−	−	1a		1a	44
21	F	13	13	0	Lesionectomy	+	+	1a	4	1*	174
22	M	5	9	4	Lesionectomy	+	−	4	4	4	137
23	M	1	2	1	ATL with H-tomy	−	−	1a	3	1	139
24	F	4	9	5	ATL with H-tomy	−	−	1a	5	5	84
25	M	0	2	2	ATL with H-tomy	−	−	1a	1a	1a	98
26	F	9	15	6	ATL	−	−	1a	1a	1a	64
27	M	1	10	9	Lesionectomy	+	+	4	1*	1*	102
28	F	3	5	2	Lesionectomy	+	+	1a	1a	1a*	97
29	M	4	27	23	ATL with H-tomy	−	−	1a	1a	1a	82
30	F	2	4	2	ATL with H-tomy	−	−	1a		1a	32
31	F	13	15	2	ATL with H-tomy	−	−	1a	1a	1a	76
32	F	3	6	3	ATL with H-tomy	−	−	1a	1a	1a	69
33	F	0	9	9	Lesionectomy	−	−	1a	1a	1a	82
34	F	15	17	2	Lesionectomy	−	−	1a		1a	24
35	F	3	10	7	ATL	+	−	2		2	48
36	F	2	4	2	Lesionectomy	+	+	3	1*	1*	68
37	M	6	26	20	ATL	−	−	1a	1a	1a	58
38	F	4	7	3	Lesionectomy	−	−	1a	1a	1a	61
39	F	1	2	1	Lesionectomy	+	−	1a		1a	50
40	M	10	23	13	Lesionectomy	+	−	1a		1a	40
41	F	0	2	2	ATL with H-tomy	−	−	1a		1a	35
42	F	18	32	14	Lesionectomy	−	−	3		1	39
43	F	4	5	1	Lesionectomy	−	−	1a		1a	29
44	M	12	13	1	Lesionectomy	+	−	1a		1a	35
45	M	6	38	32	Lesionectomy	−	−	1a		1a	31
46	F	13	17	4	ATL with H-tomy	−	−	1a		1a	12

ATL, anterior temporal lobectomy; H-tomy, hippocampectomy; LEAT, low-grade epilepsy-associated neuroepithelial tumors; F, female; M, male. *These cases underwent a second surgery for recurrent tumors during the follow-up period.

a International League Against Epilepsy outcome scale.

The identified genetic alterations among all 46 tumors were *BRAF* V600E mutation in 32 patients (69.6%), *FGFR1*-TKD duplication in 6 patients (13.0%), *FGFR1* point mutation (D650G + K654E and K636R + K654E) in 2 patients (4.3%), and *BRAF* V504_R506 duplication, *BRAF* T599dup, *BRAF* c.1802_1810delAATCTCGAT insGTC, *BRAF* V600E and *CDKN2A/B* deletion, *KIAA1549*-*BRAF* fusion, and *NF1* c3330_3333delTATG in 1 patient (2.2%) each (Table 2).

**Table 2:** Summary of genotypes, neuroimaging groups, integrated pathological diagnoses, and MNP classes in the 46 patients with LEAT.

Patient number	Genotype	Neuroimaging group	Pathological diagnosis	MNP class
1	*BRAF* V600E	1	PLNTY	NA
2	*BRAF* V600E	1	dLGG, MAPK pathway-altered	GG
3	*BRAF* V600E	1	dLGG, MAPK pathway-altered	NA
4	*FGFR1* TKD duplication	2	dLGG, MAPK pathway-altered	no match
5	*KIAA1549*-*BRAF* fusion	3	PA	no match
6	*BRAF* V600E	1	PLNTY	GG
7	*BRAF* V600E	1	dLGG, MAPK pathway-altered	NA
8	*BRAF* V600E	1	dLGG, MAPK pathway-altered	NA
9	*FGFR1* K636R + K654E	2	RGNT	DNT
10	*BRAF* V600E	1	dLGG, MAPK pathway-altered	NA
11	*BRAF* V600E	1	PXA	GG
12	*BRAF* V600E	1	dLGG, MAPK pathway-altered	GG
13	*FGFR1* TKD duplication	2	dLGG, MAPK pathway-altered	DNT
14	*BRAF* V600E	3	GG	PA
15	*BRAF* V600E	1	dLGG, MAPK pathway-altered	GG
16	*BRAF* c.1802_1810delAATCTCGATinsGTG	3	dLGG, MAPK pathway-altered	no match
17	*BRAF* V600E	1	dLGG, MAPK pathway-altered	GG
18	*BRAF* V504_R506dup	3	dLGG, MAPK pathway-altered	GG
19	*BRAF* V600E	1	GG	GG
20	BRAF V600E	3	GG	no match
21	*FGFR1* TKD duplication	2	dLGG, MAPK pathway-altered	Adult-type diffuse high-grade glioma, IDH-wildtype, subtype E
22	*FGFR1* D650G + K654E	2	RGNT	NA
23	*BRAF* V600E	1	dLGG, MAPK pathway-altered	no match
24	*BRAF* V600E	1	dLGG, MAPK pathway-altered	no match
25	*BRAF* V600E	1	GG	NA
26	*BRAF* V600E	1	GG	GG
27	*FGFR1* TKD duplication	2	dLGG, MAPK pathway-altered	DNT
28	*NF1* c3330_3333delTATG	3	dLGG, MAPK pathway-altered	GG
29	*BRAF* V600E	1	dLGG, MAPK pathway-altered	no match
30	*BRAF* V600E	1	dLGG, MAPK pathway-altered	GG
31	*BRAF* T599dup	3	GG	GG
32	*BRAF* V600E	1	GG	GG
33	*BRAF* V600E	1	dLGG, MAPK pathway-altered	GG
34	*BRAF* V600E	1	PLNTY	GG
35	*BRAF* V600E	1	GG	no match
36	*FGFR1* TKD duplication	2	dLGG, MAPK pathway-altered	DNT
37	*BRAF* V600E	1	PLNTY	GG
38	*BRAF* V600E	1	dLGG, MAPK pathway-altered	GG
39	*BRAF* V600E	1	dLGG, MAPK pathway-altered	GG
40	*BRAF* V600E	1	GG	NA
41	*BRAF* V600E	1	dLGG, MAPK pathway-altered	GG
42	*BRAF* V600E + *CDKN2A/B* deletion	3	PXA	GG
43	*FGFR1* TKD duplication	2	dLGG, MAPK pathway-altered	NA
44	*BRAF* V600E	1	GG	NA
45	*BRAF* V600E	1	dLGG, MAPK pathway-altered	NA
46	*BRAF* V600E	1	dLGG, MAPK pathway-altered*	NA

dLGG, diffuse low-grade glioma; DNT, dysembryoplastic neuroepithelial tumor; GG, ganglioglioma; LEAT, low-grade epilepsy-associated neuroepithelial tumors; MAPK, mitogen-activated protein kinase; MNP, molecular neuropathology; NA, not assessed; PA, pilocytic astrocytoma; PLNTY, pleomorphic neuroepithelial tumor of young; PXA, pleomorphic xanthoastrocytoma; RGNT, rosette-forming glioneuronal tumor; TKD, tyrosine kinase domain.

*This case was finally diagnosed as dLGG, MAPK pathway-altered, although it was accompanied by a distinct gangliocytoma component.

The calibrated score of the DNA methylation-based classification was greater than 0.9 in 26 patients. The MNP classes were GG in 20 patients, DNT in four, PA in one, and IDH-wildtype adult-type diffuse high-grade glioma subtype E in one, respectively.

The histopathological diagnoses of the 46 tumors consisted of MAPK pathway-altered dLGG in 27 patients (58.7%); GG in 10 (21.7%); PLNTY in 4 (8.7%); pleomorphic xanthoastrocytoma and rosette-forming glioneuronal tumor in 2 each (4.3% each); and pilocytic astrocytoma in 1 (2.2%; Table 1). Patient 46 was diagnosed with MAPK pathway-altered dLGG, although the tumor was accompanied by a distinct gangliocytoma component.

### 3.2 Neuroimaging features

**Figure 2:**
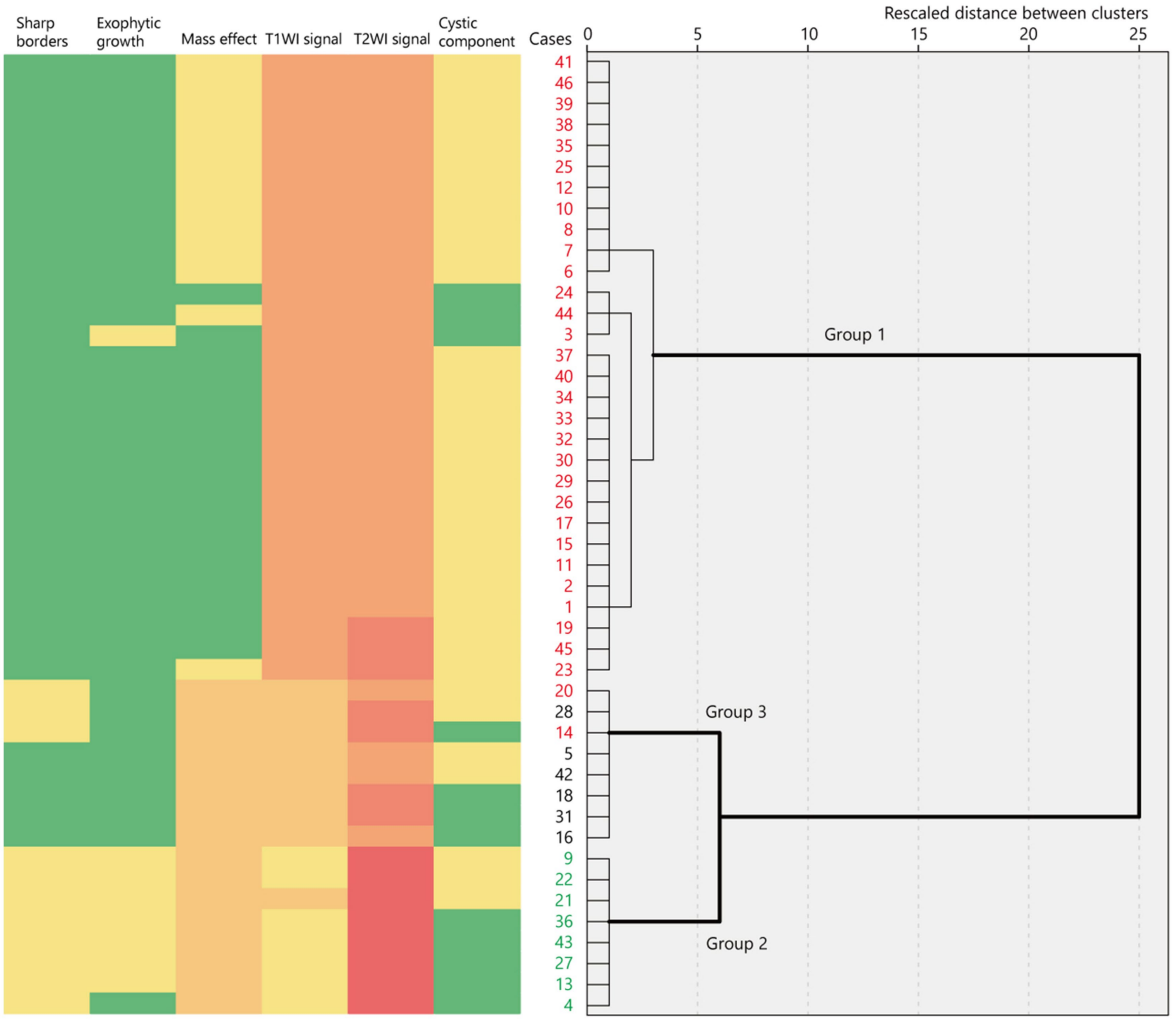
Binary or ordinary variables of neuroimaging features represented as a heatmap in 46 cases ordered using hierarchical clustering based on their rescaled distance. The dendrogram shows three major clusters of neuroimaging features. The red numbers indicate *BRAF* V600E-mutant cases while the green numbers indicate *FGFR1*-mutant cases. T1WI, T1-weighted imaging; T2WI, T2-weighted imaging.

**Figure 3:**
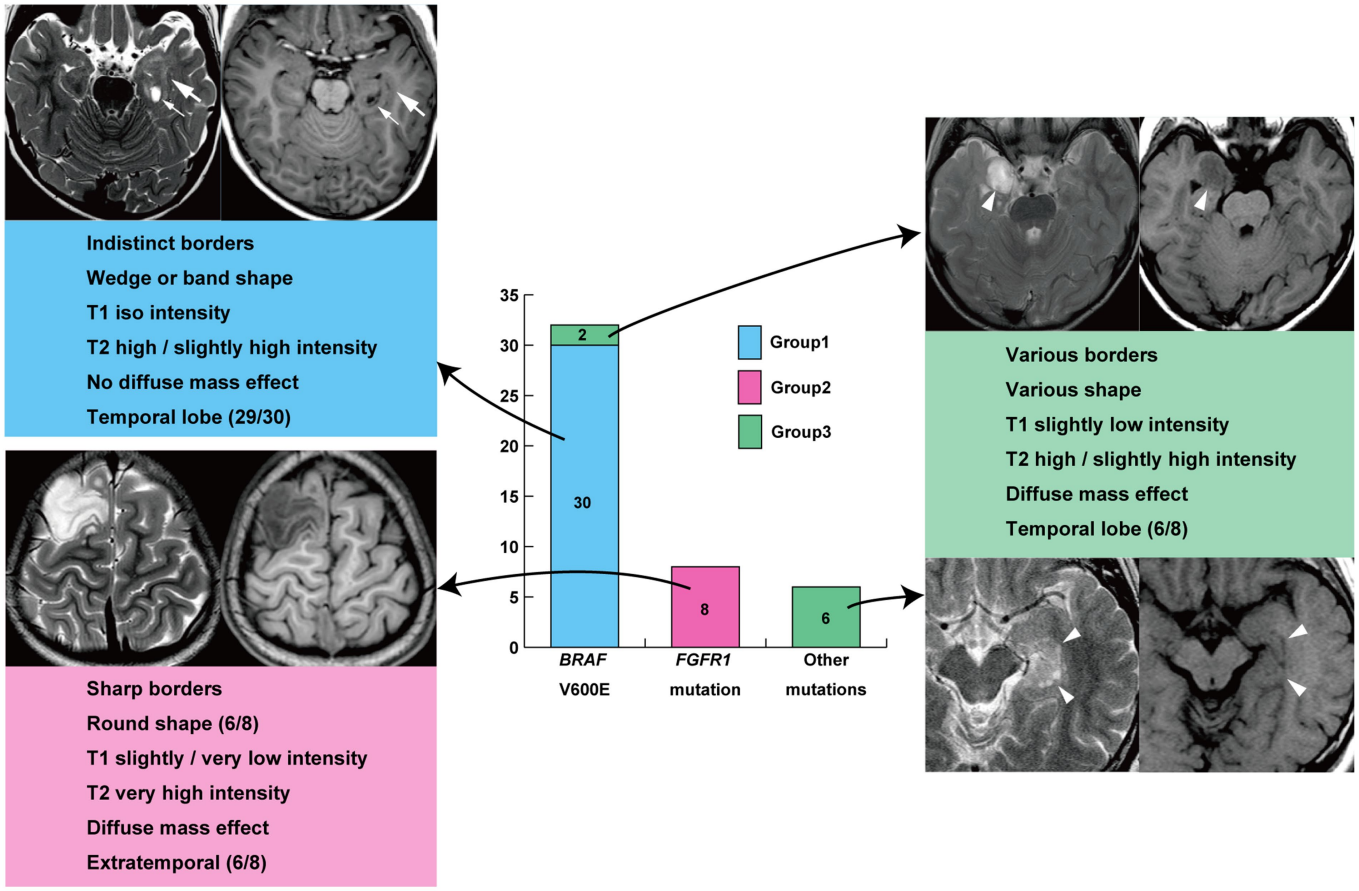
Illustrative summary of this study. T1, T1-weighted imaging; T2, T2-weighted imaging.

Hierarchical clustering analysis revealed three major groups in terms of the neuroimaging features of tumor lesions in 46 patients (Figure 2; Supplementary material). Group 1 was characterized by an indistinct tumor border and iso T1-weighted and slightly high or high T2-weighted signal intensities without a diffuse mass effect; Group 2 was characterized by a sharp tumor border and very or slightly low T1-weighted and very high T2-weighted signal intensities with a diffuse mass effect; and Group 3 included tumors with a diffuse mass effect showing slightly low T1-weighted and high or slightly high T2-weighted images. These features are illustrated in Figure 3. The Fleiss’ Kappa coefficients were 1.0 for borders, 0.897 for shapes, 1.0 for exophytic growth, 0.891 for mass effect, 1.0 for cystic component, 1.0 for hippocampal sclerosis, 1.0 for skull scalloping, 1.0 for calcification, 1.0 for FDG-PET, and 1.0 or SPECT. The intraclass correlation coefficients were 0.874 for T1-weighted images, 0.869 for T2-weighted images, 1.0 for ADC, and 1.0 for gadolinium enhancement. All coefficients were above 0.8 and the inter-rater agreement was satisfactory.

**Figure 4:**
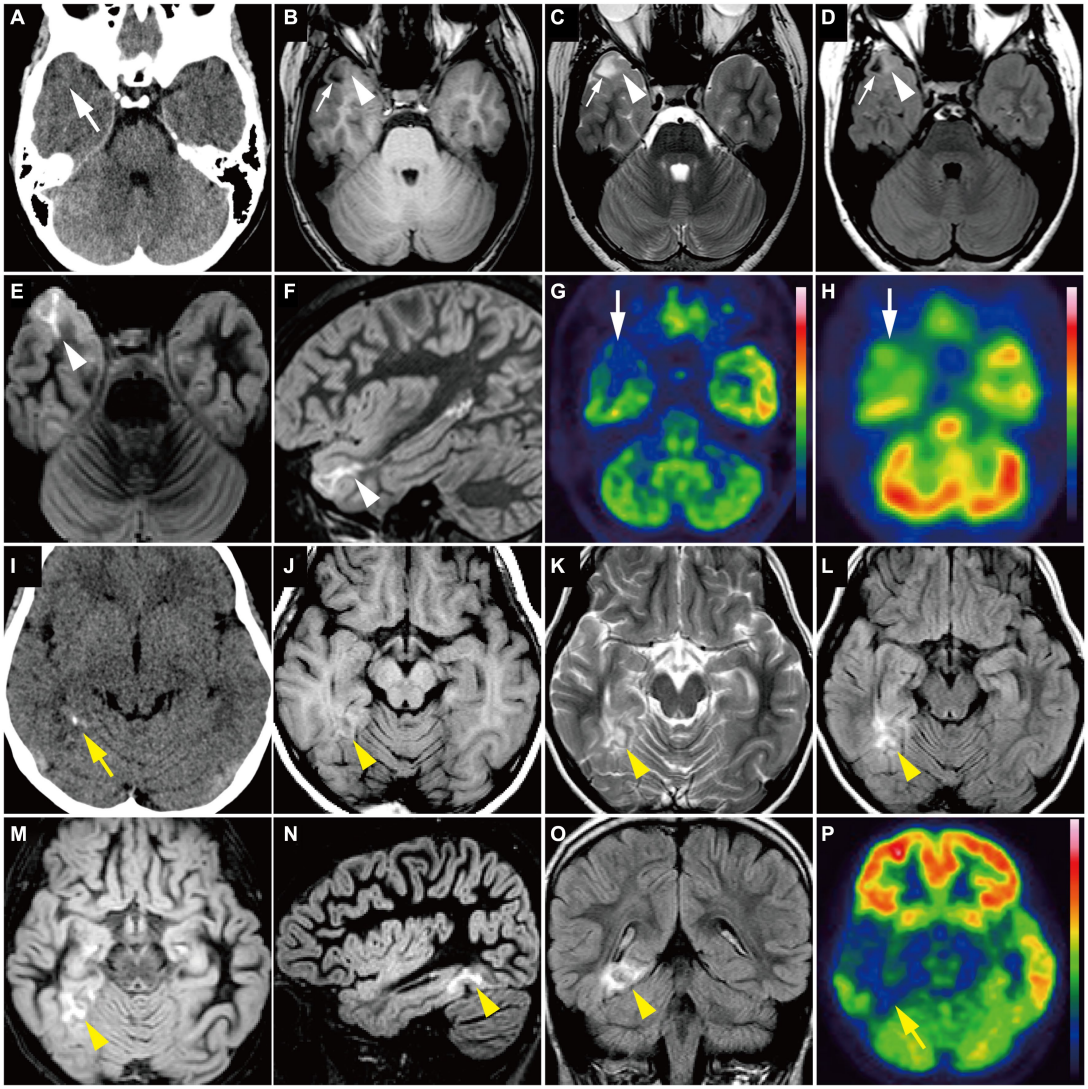
**(A–H)** A 15-year-old girl with Group 1 *BRAF* V600E-mutant low-grade epilepsy-associated neuroepithelial tumor, pathologically diagnosed as MAPK pathway-altered dLGG (Patient 26). **(A)** CT scan shows a small wedge-shaped low-density area in the right temporal tip without calcification (*white arrow*). **(B–F)** Axial T1-weighted **(B)**, axial T2-weighted **(C)**, axial FLAIR **(D)**, axial DIR **(E)**, and sagittal DIR images **(F)** demonstrate a triangular flaccid cyst in the right temporal tip (*small white arrow*s). Just medially adjacent to the cyst, an ill-defined, tiny abnormal signal area appears as iso-intensity on the T1-weighted image and slightly high intensity on the T2-weighted and FLAIR images (*white arrowheads*). Axial and sagittal DIR images show a wedge-shaped, heterogeneously high signal lesion without mass effect (*white arrowhead*s). **(G)** Axial FDG-PET image demonstrates decreased uptake in the right temporal tip (*white arrow*). *Color bar:* SUV; top = 13.00 and bottom = 0.00. **(H)** Axial ECD-SPECT image also shows decreased uptake in the same area (*white arrow*). **(I–P)** A 9-year-old girl with Group 1 *BRAF* V600E-mutant LEAT, pathologically diagnosed as MAPK pathway-altered dLGG (Patient 33). **(I)** CT scan shows a punctate calcification in the right posterior temporal lobe (*yellow arrow*). **(J–O)** Axial T1-weighted **(J)**, axial T2-weighted **(K)**, axial FLAIR **(L)**, axial DIR **(M)**, sagittal DIR images **(N)**, and coronal FLAIR **(O)** demonstrate an ill-defined, wedge-shaped tumor without mass effect located along the right medial temporal base (*yellow arrowhead*) as iso-intensity on the T1-weighted image, slightly high intensity on the T2-weighted image, and high intensity on the FLAIR and DIR images. No cystic component is noted. **(P)** Axial FDG-PET image demonstrates decreased uptake in the right temporal lobe compared with the contralateral side (*yellow arrow*). *Color bar:* SUV; top = 15.00 and bottom = 0.00. DIR, double inversion recovery; dLGG, diffuse low-grade glioma; ECD, ethyl-cysteinate-dimer; FDG, fluorodeoxyglucose; FLAIR, fluid-attenuated inversion recovery; LEAT, low-grade epilepsy-associated neuroepithelial tumor; MAPK, mitogen-activated protein kinase; PET, positron emission tomography; SPECT, single photon emission computed tomography; SUV, standardized uptake value.

Group 1 tumors were identified in 30 patients (65.2%), all but one of which were located in the temporal lobe, specifically in the mesial (*n* = 20) and lateral temporal lobes (*n* = 9). The tumors were localized along the temporal base in 14 patients. One tumor (patient 19) was located in the eloquent area (visual area). The mean tumor volume was 13,163 ± 10,069 mm^3^ (792–39,312 mm^3^). Tumor shape was wedge (Figure 4; *n* = 11) or band (Supplementary material; *n* = 19). No mass effect was observed in 17 patients, but a focal mass effect (Supplementary material) was found in 13 patients. Signal intensity appeared isointense on T1-weighted images, slightly high or high on T2-weighted images, and slightly high on the ADC map. No contrast enhancement was found in 13 of 17 examined patients, but faint enhancement was observed in a portion of the tumor mass in four patients. Additionally, 26 patients had flaccid cystic components (Figures 4B–D; Supplementary material), and three had hippocampal sclerosis. None of the patients developed skull scalloping. Calcifications were seen in 18 patients (Figure 4I; Supplementary material), and 12 of the 13 patients with focal mass effect had associated calcifications (Supplementary material). All patients had cystic components or calcifications or both, except Patient 24. FDG-PET hypometabolism was observed in the tumor area in all examined patients (Figures 4G,P; Supplementary material). Ethyl-cysteinate-dimer-SPECT revealed lower regional CBF (rCBF) in all but one of the examined patients (Figure 4H).

**Figure 5:**
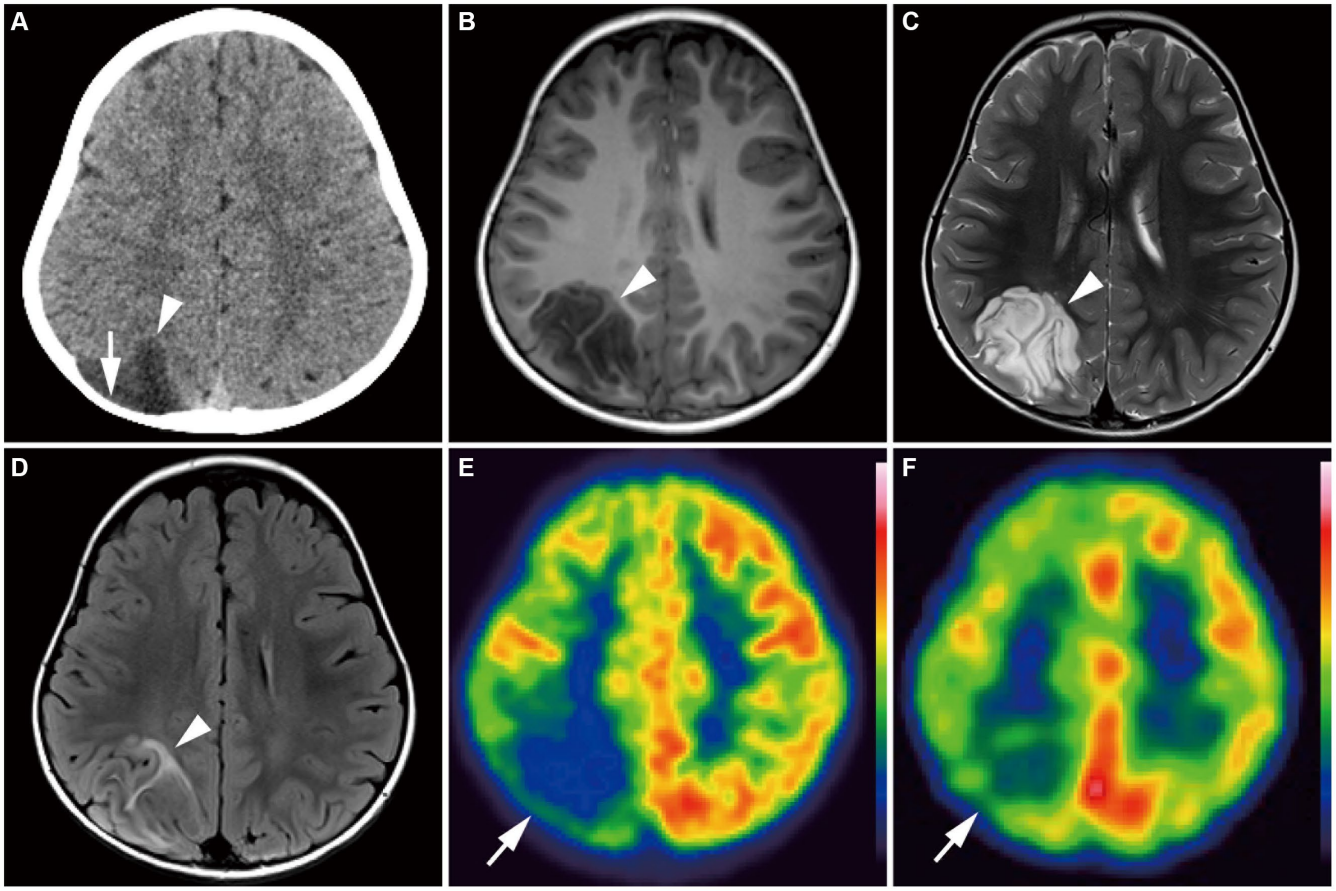
A 4-year-old girl with Group 2 *FGFR1*-mutant low-grade epilepsy-associated neuroepithelial tumors, pathologically diagnosed as MAPK pathway-altered dLGG (Patient 36). **(A)** CT scan shows an exophytic very low-density mass in the right parietal lobe (*arrowhead*) with skull scalloping (*arrow*). **(B–D)** Axial T1-weighted **(B)**, axial T2-weighted **(C)**, and axial FLAIR images **(D)** show a well-defined, round-shaped mass in the right parietal lobe appearing as very low intensity on the T1-weighted image, very high intensity on the T2-weighted image, and a hyperintense rim with a heterogeneous inner component on the FLAIR image (*arrowhead*). **(E)** Axial FDG-PET image demonstrates decreased uptake in the right hemisphere, especially in the parietal lobe (*arrow*). *Color bar:* standardized uptake values; top = 7.00 and bottom = 0.00. **(F)** Axial ECD-SPECT image also shows decreased uptake in the same area (*arrow*). dLGG, diffuse low-grade glioma; ECD, ethyl-cysteinate-dimer; FDG, fluorodeoxyglucose; MAPK, mitogen-activated protein kinase; FLAIR, fluid-attenuated inversion recovery; PET, positron emission tomography; SPECT, single photon emission computed tomography.

Group 2 tumors were identified in eight patients (17.4%; Figure 5). The tumors were located in extratemporal lobes in six patients, specifically in the frontal (*n* = 4), parietal (*n* = 1), and parieto-occipital (*n* = 1) lobes. Two tumors were located in the eloquent area (motor area in patient 27 and visual area in patient 36). The mean tumor volume was 70,982 ± 44,198 mm^3^ (5,472–165,620 mm^3^), larger than that of Group 1 (*p* = 0.01). The tumor was associated with sharp borders and a diffuse mass effect in all 8 patients. Six patients had round tumors, and six had exophytic growth. Tumor intensity was very low or slightly low on T1-weighted images, very high on T2-weighted images, and very high on the ADC map, characterized by heterogeneous inner components (Figures 5B–D). Six of the seven examined patients showed no contrast enhancement. A portion of the mass was faintly enhanced in one patient (Patient 9). Hippocampal sclerosis was not suggested in any patients. Three patients had skull scalloping (Figure 5A), and two had calcifications. Hypometabolism on FDG-PET and decreased rCBF on ethyl-cysteinate-dimer-SPECT were observed in all examined patients (Figures 5E,F).

**Figure 6:**
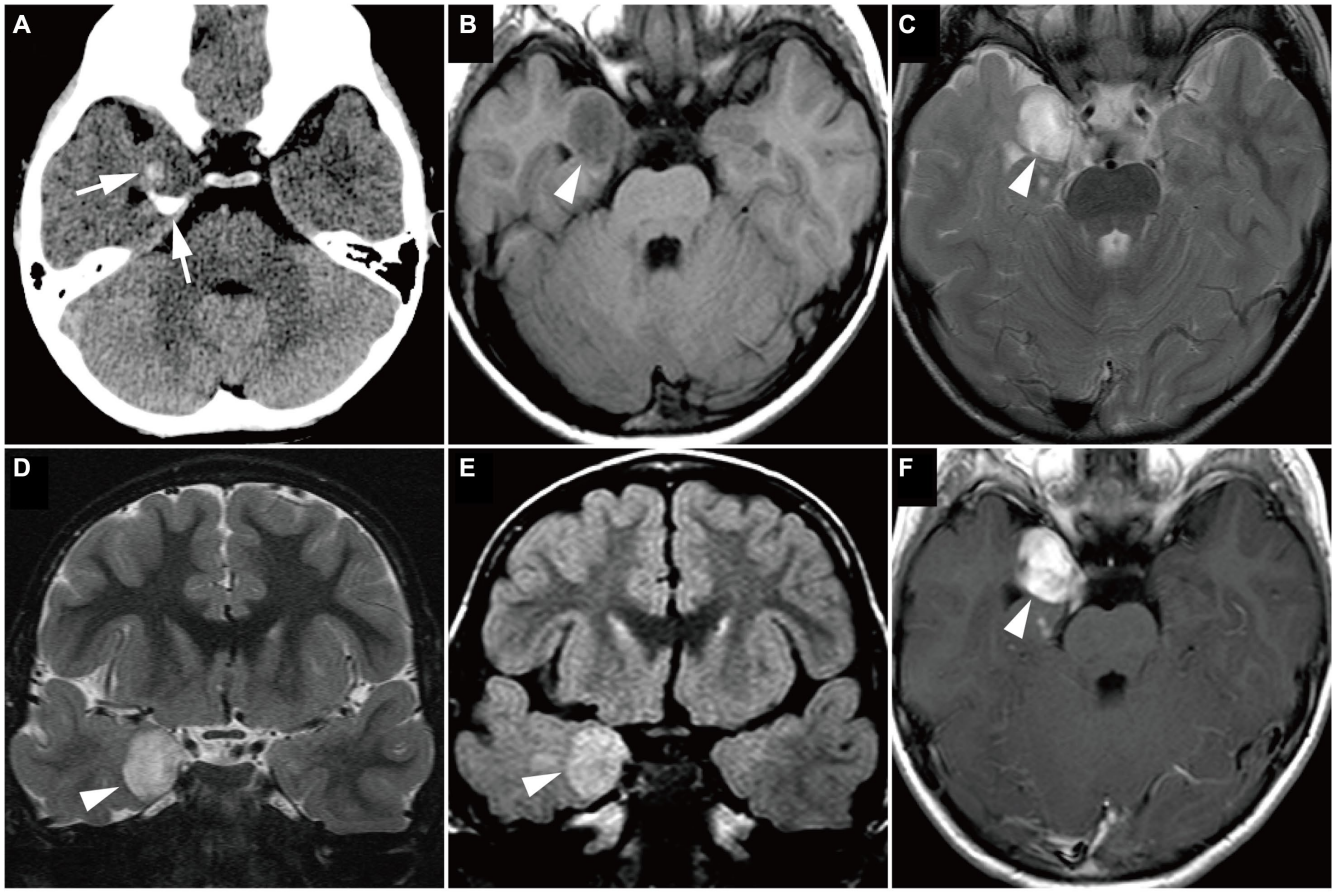
Magnetic resonance imaging image of a Group 3 tumor with *BRAF* V600E mutation, pathologically diagnosed as ganglioglioma in a 4-year-old boy (Case 14). **(A)** Computed tomography scan shows a small, dense, crescent-shaped calcification in the right medial temporal lobe (white arrows). **(B–F)** Axial T1-weighted **(B)**, axial T2-weighted **(C)**, coronal T2-weighted **(D)**, coronal FLAIR **(E)**, and axial gadolinium-enhancement T1-weighted **(F)** images show a well-defined, round mass in the right medial temporal lobe (white arrowheads), with slightly low intensity on the T1-weighted image, high intensity on the T2-weighted image, and high intensity on the FLAIR image. The mass is uniformly enhanced. FLAIR, fluid-attenuated inversion recovery.

The other tumors in the eight remaining patients (14.6%) were classified as Group 3 (Figure 6; Supplementary material). All tumors were characterized by a diffuse mass effect without exophytic growth. Signal intensity was slightly low on T1-weighted images, high or slightly high on T2-weighted images, and slightly high on the ADC map. Homogeneous gadolinium enhancement was observed in four of the six examined patients. The tumors were located in the mesial temporal region in six patients. No tumors were located in the eloquent area.

### 3.3 Association of genotypes with neuroimaging groups, histopathological diagnoses

All 30 tumors in Group 1 were associated with *BRAF* V600E mutations, and all 8 tumors in Group 2 were associated with *FGFR1* mutations. Group 3 tumors included two cases of *BRAF* V600E mutation and one of *BRAF* V600E mutation co-occurring with *CDKN2A/B* deletion; four of the other five cases were *BRAF*-related gene alterations. Thus, Group 1 neuroimaging features had a sensitivity of 93.8% and specificity of 100% for *BRAF* V600E mutation (Table 3). Group 2 neuroimaging features exhibited a sensitivity and specificity of 100% for *FGFR1* mutations in our cohort.

**Table 3:** Association of genotypes with neuroimaging groups and pathological diagnoses in 46 patients with LEAT.

Genotypes	Neuroimaging group			Histopathological diagnoses					
	1	2	3	dLGG, MAPK pathway-altered	GG	PLNTY	PXA	RGNT	PA
*BRAF* V600E-mutant	30	0	2	18	9	4	1	0	0
*FGFR1-*mutant	0	8	0	6	0	0	0	2	0
Others	0	0	6	3	1	0	1	0	1

dLGG, diffuse low-grade glioma; GG, ganglioglioma; LEAT, low-grade epilepsy-associated neuroepithelial tumors; MAPK, mitogen-activated protein kinase; PA, pilocytic astrocytoma; PLNTY, pleomorphic neuroepithelial tumor of young; PXA, pleomorphic xanthoastrocytoma; RGNT, rosette-forming glioneuronal tumor.

The pathological diagnosis of MAPK pathway-altered dLGG was not associated with a specific genotype (Table 3). MAPK pathway-altered dLGG (*n* = 27) had a sensitivity of 60.0% and specificity of 43.8% for *BRAF* V600E mutations and a sensitivity of 75.0% and specificity of 44.7% for *FGFR1* mutations. GG (*n* = 10) had a sensitivity of 92.9% and specificity of 28.1% for *BRAF* V600E mutations. PLNTY (*n* = 4) had a sensitivity of 100% and specificity of 12.5% for *BRAF* V600E mutations.

### 3.4 Association of molecular neuropathology classes with neuroimaging groups

MNP showed that all Group 1 tumors (*n* = 16) were classified as GG. Four out of five Group 2 tumors were classified as DNT and one was classified as IDH-wildtype adult-type diffuse high-grade glioma subtype E. Four out of five Group 3 tumors were classified as GG and one was classified as PA. In the case of *BRAF* V600E-mutant LEAT, all 16 tumors with Group 1 imaging features were classified as GG and one tumor with Group 3 imaging features (Case 14, Figure 6) was classified as pilocytic astrocytoma (Tables 2, 4).

**Table 4:** Association of neuroimaging groups and MNP classes in 17 *BRAF* V600E-mutant LEAT.

Neuroimaging groups	MNP class	
	GG	PA
Group 1	16	0
Group 3	0	1

LEAT, low-grade epilepsy-associated neuroepithelial tumors; MNP, molecular neuropathology; GG, ganglioglioma; PA, pilocytic astrocytoma.

### 3.5 Association of genotypes, neuroimaging groups, and pathological diagnoses with postsurgical outcome

**Figure 7:**
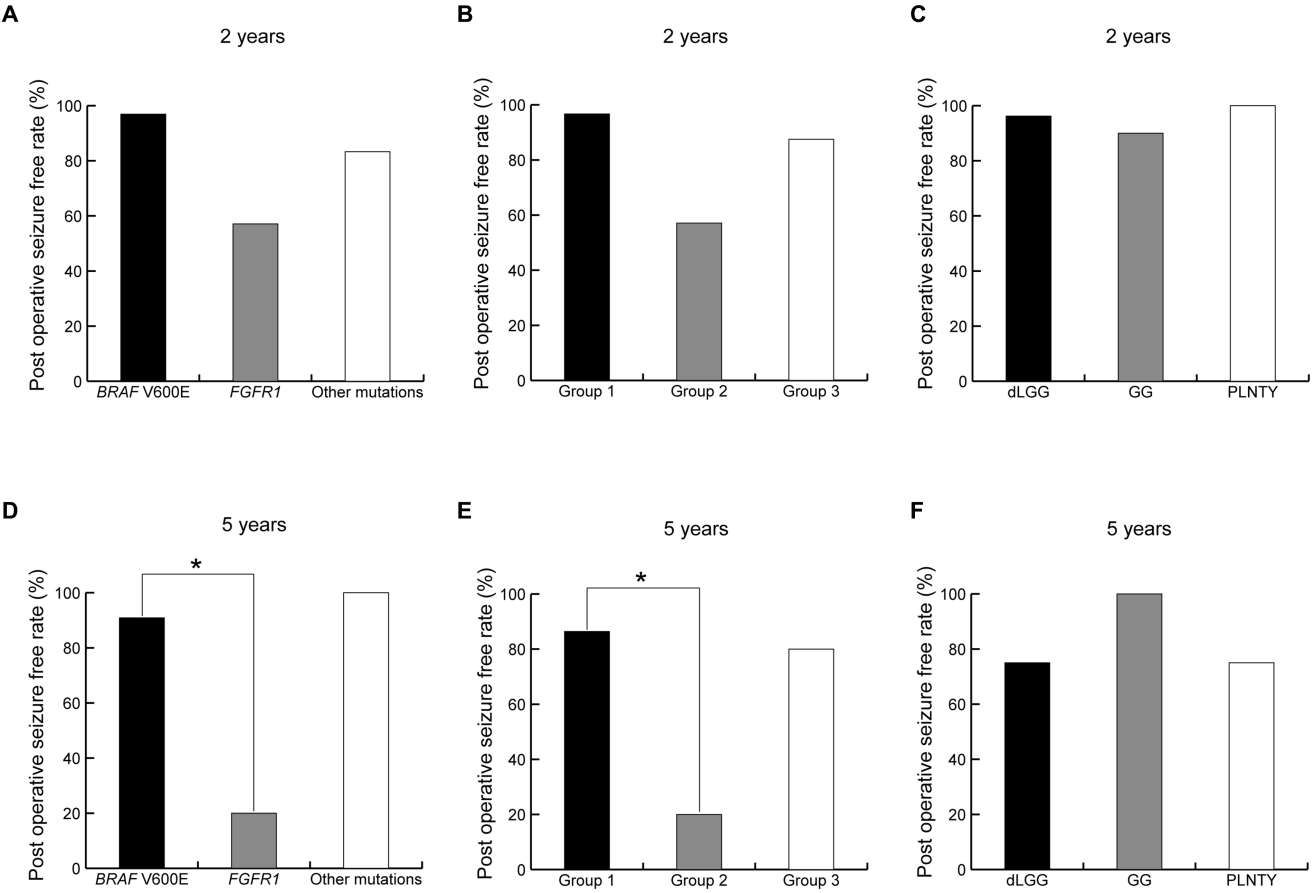
Difference of seizure freedom ratio at 2 and 5 years after surgery between genotypes **(A,D)**, neuroimaging groups **(B,E)** and pathological diagnoses **(C,F)**. *indicates a statistically significant difference. dLGG, diffuse low-grade glioma, MAPK pathway altered; GG, ganglioglioma; PLNTY, pleomorphic neuroepithelial tumor of young.

The complete seizure-free rates in patients with *BRAF* V600E, *FGFR1*, and the other mutations were 96.9% (31/32), 57.1% (4/7), and 83.3% (5/6) at 2 years postoperatively (Figure 7A) and 90.9% (20/22), 20.0% (1/5), and 100% (4/4) at 5 years postoperatively, respectively (Figure 7D). The complete seizure-free rates in patients with Group 1, 2, and 3 imaging features were 96.7% (29/30), 57.1% (4/7), and 87.5% (7/8) at 2 years postoperatively (Figure 7B) and 86.4% (19/22), 20.0% (1/5), and 80.0% (4/5) at 5 years postoperatively, respectively (Figure 7E). The rates at 5 years postoperatively were higher in patients with *BRAF* V600E mutations than in those with *FGFR1* mutations (*p* = 0.02), and similarly in patients with Group 1 imaging features compared with patients with Group 2 features (*p* = 0.04). The complete seizure-free rates in patients with MAPK pathway-altered dLGG; GG; and PLNTY were 96.2% (25/26), 90.0% (9/10), and 100% (4/4) at 2 years postoperatively (Figure 7C) and 75.0% (15/20), 100% (6/6), and 75% (3/4) at 5 years postoperatively (Figure 7F), with no significant difference between the pathological diagnoses.

Tumor recurrence or regrowth was observed in seven patients at a significantly higher rate for *FGFR1* mutations (50.0%, 4/8) than *BRAF* V600E mutations (6.3%, 2/32; *p* = 0.048). All seven patients were pathologically diagnosed with MAPK pathway-altered dLGG.

## 4 Discussion

This single-hospital study revealed two distinct genotype-relevant neuroimaging features among 46 LEAT carrying identified genetic alterations: Group 1 neuroimaging features with *BRAF* V600E mutations, which were the most common, and Group 2 neuroimaging features with *FGFR1* mutations. Group 1 neuroimaging features were observed in 93.8% (30/32) of *BRAF* V600E-mutant tumors, whereas all *FGFR1*-mutant tumors showed Group 2 features. The neuroimaging features of Group 1, such as ill-defined border, wedge/band-shape, and iso T1 and slightly high or high T2-intensity lesions without mass effect or enhancement, may resemble those of focal cortical dysplasia, at least in part. However, most contained flaccid cystic components, and some showed a focal mass effect, gadolinium enhancement, and calcifications; these features were distinct from those of focal cortical dysplasia. The neuroimaging features of Group 2, such as well-defined and very or slightly low T1- and very high T2-intensity exophytic growing masses, resembled those of DNT. However, our genotype–phenotype integrated diagnoses based on the 2021 WHO classification of CNS tumors were not as relevant to the genotypes as neuroimaging features; the most frequent diagnosis was MAPK pathway-altered dLGG in both *BRAF* V600E and *FGFR1* mutations. The diagnosis of GG and PLNTY was relatively specific to *BRAF* V600E mutations. One reason for the radiological-to-genotype correlation is that neuroimaging can evaluate the entire tumor characteristics, whereas pathological examination may evaluate only a small portion of the polymorphous tumor. The present study also revealed a higher chance of postoperative seizure freedom in patients with Group 1 *BRAF* V600E-mutant tumors than in those with Group 2 *FGFR1*-mutant tumors. We believe that these neuroimaging subtypes are useful to infer the genotype and postoperative seizure outcome before surgery because somatic mutations in the tumor cannot be diagnosed before surgery. Furthermore, differences in MNP classes between BRAF V600E-mutant Group 1 and Group 3 tumors suggest that the same genetic mutation can result in different neuroimaging phenotypes depending on the methylation class.

Neoplasms with similar neuroimaging features as our Group 1 *BRAF* V600E-mutant LEAT may have been radiologically and pathologically diagnosed under various names, including GG, low-grade glioma, DNT, diffuse glioneuronal tumor, PLNTY, or even focal cortical dysplasia, because of their polymorphous histologic features or limited amount of resection specimens or both (5, 7, 15–17). We believe that Group 1 *BRAF* V600E-mutant tumor represents a previously unrecognized cluster with a strong association between neuroimaging features and genotype in LEAT, irrespective of histopathological and integrated diagnoses. The neuroimaging appearance of GG is reportedly variable, often displaying a mix of solid and cystic components; some display an indistinct border like Group 1 tumors (18). Low-grade gliomas can also appear similar to Group 1 tumors but are typically homogeneous with low T1-weighted and high T2-weighted signals (19). Al-Hajri et al. reported an association between pathological diagnosis and radiological features in 27 patients with LEAT, including 13 patients with diffuse glioneuronal tumor (6). MRI findings of these diffuse glioneuronal tumor cases were very similar to those of our Group 1 *BRAF* V600E-mutant LEAT. The neuroimaging features of *BRAF* V600E-mutated PLNTY have been demonstrated previously (20, 21). Wedge-shaped lesions with subcortical low T1 and high T2 intensity, suggestive of a flaccid cystic component, and without mass effect (22) are very similar to our Group 1 tumors.

Group 2 *FGFR1*-mutant tumors have probably been interpreted as DNT on imaging. The 2021 WHO Classification of Tumors of the CNS included two entities of DNT, i.e., simple- and complex-form DNT (9). The neuroimaging features of DNT used to be classified as follows: Type 1, cystic/polycystic-like, well-defined, and strongly hypointense on T1-weighted images; Type 2, nodular-like, with heterogeneous intensity; and Type 3, dysplastic-like, iso/hypointense on T1-weighted images, poor delineation, and gray-white matter blurring (22). Group 2 neuroimaging features in our series correspond to Type 1 in their description, which is associated with simple- and complex-form DNTs (9, 23). *FGFR1*-mutant tumors have been pathologically diagnosed as DNT, rosette-forming glioneuronal tumor, low-grade glioma, and GG (5, 24). Type 3 imaging features in DNT are also similar to those of our Group 1 tumors.

Group 3 tumors do not share common genotype or pathological features, although seven of the eight cases had *BRAF*-related gene alterations. This result suggests that mutations in the *BRAF* gene, other than V600E, cause various neuroimaging phenotypes. Notably, two cases of *BRAF* V600E mutation (Patients 14 and 20) were similar to each other (Figure 6) but had imaging features distinct from those of Group 1 tumors. Our MNP analysis suggest that epigenetic differences including the DNA methylation profile may cause different imaging phenotypes in *BRAF* V600E-mutant LEAT (Table 4).

The fact that Group 1 *BRAF* V600E-mutant tumors and Group 2 *FGFR1*-mutant tumors have not been recognized as distinct groups of tumors may be owing to the difficulty of pathological diagnoses in LEAT (12). The concordance of pathological diagnosis between GG and DNT is as low as 40%, even among experts. We speculate that tumors like those observed in our Group 1 have often been associated with the histopathological diagnosis of GG and Group 2 tumors with DNT. The pathological diagnoses of GG and PLNTY occurred mostly in tumors with Group 1 neuroimaging features and *BRAF* V600E mutation. None of our cases were histopathologically diagnosed as DNT, but the neuroimaging features of Group 2 tumors were characteristic of what has been considered DNT, as discussed above. However, the majority of Group 1 and 2 tumors were diagnosed as MAPK pathway-altered dLGG based on the WHO 2021 classification in our study, without a clear association with the genotypes.

Our results suggest that MAPK pathway-altered dLGG is a common histopathological diagnosis of LEAT, but the differentiation between dLGG, GG, and DNT is debatable. The diagnosis of MAPK pathway-altered dLGG seemed more appropriate than GG in our study when ganglion cells were not definitively identified in the histological evaluation and genetic information was provided. Similarly, the diagnosis of MAPK pathway-altered dLGG seemed more appropriate than DNT when specific glioneuronal elements were not definitively observed. The agreement between experts on the diagnosis of GG and DNT becomes low when specific histological features are not observed. “Diffuse glioneuronal tumor” was proposed as an umbrella term for the difficult-to-classify CD34-expressing tumors that lack the specific histologic findings of GG or DNT (6).

We observed different postoperative outcomes between the neuroimaging phenotypes in LEAT, suggesting the usefulness of our genotype-specific neuroimaging classification for planning surgery and predicting the outcomes of patients with LEAT. The rate of seizure freedom was higher in Group 1 *BRAF* V600E-mutant tumors than in Group 2 *FGFR1*-mutant tumors. Tumor recurrence was more frequent in *FGFR1*-mutant tumors than in *BRAF* V600E-mutant tumors. Tumor recurrence is likely associated with recurrent seizures. No previous studies have reported the difference in the postoperative outcomes between different genotypes in LEAT. Several studies have reported that gross total resection is the main factor for seizure freedom in patients with LEAT (25, 26). The unfavorable seizure outcome in *FGFR1*-mutant LEAT is presumably attributed to the presence of residual tumor, because of the larger volume of *FGFR1*-mutant LEAT than those of other genotypes, as well as the different biological natures of *FGFR1*-mutant tumors and other genotypes. Coexisting hippocampal sclerosis, known as dual pathology, and additional removal of the hippocampus may be factors affecting the seizure outcome. This study included only three patients with hippocampal sclerosis; this number was too small for statistical evaluation. The tumor location may be a confounding factor for the postoperative seizure outcome because temporal lobe resection is an independent prognostic factor for a better outcome (27). In this study, Group 1 tumors exclusively occurred in the temporal lobe, while most of the Group 2 tumors occurred in the extratemporal region. Tumors were located in the eloquent areas in 3 patients. Five patients underwent reoperation for residual tumors. The tumor location in these patients was often extratemporal, with two cases in the medial temporal lobe, one in the frontal lobe-insula, one in the temporal-occipital lobe, and one in the parietal lobe. The tumor location and its vicinity to the eloquent area can influence on the tumor removal rate and seizure outcome. Epilepsy duration can also be the predictor of post-surgical seizure freedom (28). Factors for seizure outcome need to be further examined with a larger number of patients, including the clinical factors mentioned above.

The most significant feature of our study is that the study population consisted of patients with drug-resistant epilepsy consecutively recruited from a single tertiary epilepsy center. A previous single-center study identified the genetic alterations in 32 low-grade glioneuronal, but not glial, tumors, including *BRAF* V600E mutations (*n* = 13, 41%), *FGFR1* mutations (*n* = 9, 28%), and other mutations (*n* = 10, 32%), but no *MYB/MYBL1* mutations (8). This range of mutation types is similar to that observed in our study but differs from that observed in other studies (5, 7). A previous multicenter study (7) of 56 low-grade neuroepithelial tumors identified 35 tumors in the cerebral cortex carrying *FGFR1* mutations (*n* = 10, 29%), *BRAF* mutations (*n* = 7, 20%), and *MYB/MYBL1* alterations (*n* = 9, 26%), and another multicenter series of 91 low-grade neuroepithelial tumors reported *FGFR1* mutations (*n* = 30, 33%), *BRAF* V600E mutations (*n* = 10, 11%), *MYB/MYBL1* alterations (*n* = 22, 24%), and other mutations (*n* = 29, 32%) (5). These previous studies included surgical specimens from patients with and without epilepsy, which may explain the difference in the ranges of mutation types.

This study has some limitations. First, there may have been patient selection bias because genetic mutations were not identified in approximately 30% of the 76 patients with LEAT who were excluded from the present study. Therefore, further investigations are desirable, including RNA sequencing, whole-genome analysis, and methylation analysis. In addition, as our cohort was selected from a single epilepsy center, a multicenter study with a larger number of epilepsy patients is needed in the future. Finally, a more advanced investigation of the pathology of LEAT is warranted.

In conclusion, there are two major genotype-relevant neuroimaging subtypes in LEAT: (1) ill-defined, wedge/band-shaped, iso T1 intensity and slightly high or high T2 intensity lesions carrying *BRAF* V600E mutations and (2) well-defined, very or slightly low T1- and very high T2-intensity exophytic growing masses carrying *FGFR1* mutations. The latter tumor was associated with a higher risk of tumor and seizure recurrence. Accordingly, we propose a new neuroimaging classification of LEAT that differentiates tumors with *BRAF* V600E or *FGFR1* mutations, which are relevant to postsurgical seizure outcomes. Our results will likely contribute to optimal patient care, including accurate preoperative differential diagnoses, appropriate classification of LEAT, and future application of molecular targeted drugs.

## Data availability statement

The raw data supporting the conclusions of this article will be made available by the authors, without undue reservation.

## Ethics statement

The studies involving humans were approved by Ethics committee of the National Center of Neurology and Psychiatry, Japan. The studies were conducted in accordance with the local legislation and institutional requirements. Written informed consent for participation in this study was provided by the participants’ legal guardians/next of kin.

## Author contributions

KI: Conceptualization, Data curation, Formal analysis, Funding acquisition, Investigation, Methodology, Project administration, Resources, Validation, Visualization, Writing – original draft, Writing – review & editing. HF: Data curation, Formal analysis, Investigation, Validation, Writing – review & editing. FS: Formal analysis, Methodology, Validation, Writing – review & editing. KM: Data curation, Formal analysis, Investigation, Methodology, Validation, Writing – review & editing. Y-iG: Conceptualization, Formal analysis, Funding acquisition, Investigation, Methodology, Supervision, Writing – review & editing. YS: Conceptualization, Data curation, Investigation, Methodology, Supervision, Validation, Writing – review & editing. TS: Data curation, Formal analysis, Investigation, Methodology, Writing – review & editing. HyS: Conceptualization, Data curation, Formal analysis, Investigation, Methodology, Validation, Writing – review & editing. HM: Conceptualization, Data curation, Formal analysis, Funding acquisition, Investigation, Methodology, Supervision, Validation, Writing – review & editing. YK: Data curation, Formal analysis, Investigation, Methodology, Validation, Writing – review & editing. TN: Formal analysis, Investigation, Methodology, Writing – review & editing. HmS: Formal analysis, Investigation, Methodology, Supervision, Writing – review & editing. MI: Conceptualization, Formal analysis, Funding acquisition, Investigation, Methodology, Project administration, Resources, Supervision, Validation, Visualization, Writing – original draft, Writing – review & editing. NS: Conceptualization, Data curation, Formal analysis, Funding acquisition, Investigation, Methodology, Supervision, Validation, Writing – original draft, Writing – review & editing.

## Footnotes

^1^https://www.molecularneuropathology.org/mnp/
