# Supplementary material for: Genotype-relevant neuroimaging features in low-grade epilepsy-associated tumors
Source: Front Neurol. 2024 Jul 16;15:1419104. doi: 10.3389/fneur.2024.1419104 (PMC11286587; doi:10.3389/fneur.2024.1419104)
Supplement: Supplementary file 1 [file Data_Sheet_1.docx]

Supplementary Material

# Supplementary Methods

## DNA preparation from frozen tissue

DNA was extracted from frozen archival material using a QIAamp DNA Mini kit (Qiagen, Hilden, Germany) according to the manufacturer’s instructions. The extracted DNA was concentrated using a QIAamp DNA Micro kit (Qiagen) and then eluted in TE0.1 buffer (10 mM Tris-HCl, pH 8.0, and 0.1 mM EDTA). DNA concentration and quality were confirmed using a NanoDrop ONE spectrophotometer and the Qubit dsDNA HR Assay Kit (Thermo Fisher Scientific, Waltham, MA, USA).

## DNA extraction from formalin-fixed paraffin-embedded (FFPE) tissue

DNA extraction from two or three 10-μm paraffin sections was performed according to the in-house one-tube FFPE method as described previously (1) with a minor modification, i.e., the use of Triton X-100 instead of NP40 in the one-tube FFPE buffer. The crude lysate was centrifuged for 15 min at 14,000 rpm. The supernatant was purified with the QIAamp DNA Micro kit, and DNA quality was confirmed as described above.

## Image acquisition

Preoperative interictal fluorine-18-fluorodeoxyglucose ([^18^F]FDG)-PET and interictal technetium-99m-ethyl-cysteinate-dimer SPECT (99mTc- ethyl-cysteinate-dimer [ECD]-SPECT) were performed within 1 year before surgery in 24 and 17 patients, respectively. A combined 16-slice PET/CT scanner was used for FDG-PET. The patients had fasted for >6 h, and the blood glucose levels were measured, followed by an intravenous injection of 4–6 MBq/kg [^18^F]FDG 40 min before brain PET/CT started.

The following settings were used for MR images. Axial or coronal T1-weighted imaging: repetition time (TR)=550–624 ms, echo time (TE)=10–15 ms, flip angle = 75°–90°, field of view (FOV)=220–230 × 228–239 mm, matrix=169–202 × 240–256, 3- to 5-mm thickness, number of excitations (NEX)=1 or 2. Three-dimensional T1-weighted imaging: TR = 7.2–2,080 ms, TE=2.25–3.93 ms, flip angle=9°–15°, voxel size=0.8 × 0.8 × 0.8–1.2 × 1.2 × 1.2 mm^3^, FOV=250–315 × 259–327 mm, matrix=192–288 × 256–320, NEX=1. Axial or coronal T2-weighted imaging: TR=3,600–9,000 ms, TE=80–101 ms, flip angle=90°–180°, FOV=173–230 × 180–239 mm, matrix=179–348 × 256–512, 2- to 5-mm thickness, NEX=1 or 2. Axial or coronal FLAIR imaging: TR=9,000–12,000 ms, inversion time (TI)=2,500–2,700 ms, TE=90–120 ms, flip angle=90°–170°, FOV=212–230 × 220–239 mm, matrix=168–202 × 256–320, 3- to 5-mm thickness, NEX=1 or 2. Three-dimensional FLAIR imaging: TR=4,700–5,000 ms, TI=1,600–1,800 ms, TE=291–413 ms, flip angle=90°–120°, voxel size=1.0 × 1.0 × 1.0 or 1.1 × 1.1 × 1.1 mm^3^, FOV=250–260 × 250–260 mm, matrix=240–256 × 240–256, NEX=1 or 2. Diffusion-weighted imaging: TR=3,500–8,800 ms, TE=53–113 ms, flip angle=90°, 3- to 5-mm thickness, FOV=220–230 × 229–239 mm, matrix=96–128 × 128, NEX=1 or 2. Diffusion was measured along 3, 12, 15, or 32 non-collinear directions using a diffusion-weighted factor b of 0 and 1000 s/mm^2^ or 0, 1,000, and 2,000 s/mm^2^. Three-dimensional DIR imaging: TR=5,500–7,500 ms, long TI/short TI=450/2,550–3,000 ms, TE=252–316 ms, flip angle=90°–120°, FOV=250 × 250 mm, matrix=208–256 × 208–256, 3- to 5-mm thickness, NEX=1 or 2.

A standard PET/CT bed with a built-in head holder was used for the emission scans (15 min/bed position; matrix, 336 × 336; pixel size, 0.89 × 0.89 mm) of the brain PET/CT protocol (one bed position; FOV, 30.0 cm axial) in three-dimensional mode. SPECT or SPECT/CT scanners with a two-head rotating gamma camera system with a low-energy high-resolution collimator and the following parameter settings were used to acquire the SPECT data: photo peak centered on 140 keV and acceptance window of 20%, 30 projections per head over 180° on a 128 × 128 matrix, and acquisition time of approximately 20 min. A Butterworth filter (cutoff frequency, 0.5) was used to filter the projections, which were reconstructed by the filtered back-projection method in trans-axial slices parallel to the orbitomeatal line and parallel to the long axis of the temporal lobe to produce coronal sections. Attenuation correction was performed with the Chang method for SPECT and the CT scans for SPECT/CT (2).

**References**

Atanesyan L, Steenkamer MJ, Horstman A, Moelans CB, Schouten JP, Savola SP. Optimal fixation conditions and DNA extraction methods for MLPA analysis on FFPE tissue-derived DNA. Am J Clin Pathol. (2017) 147:60–8. doi: 10.1093/ajcp/aqw205

Chang LT. A method for attenuation correction in radionuclide computed tomography. IEEE Trans Nucl Sci (1978) 25:638–43. doi: [10.1109/TNS.1978.4329385](https://doi.org/10.1109/TNS.1978.4329385)

# Supplementary figures


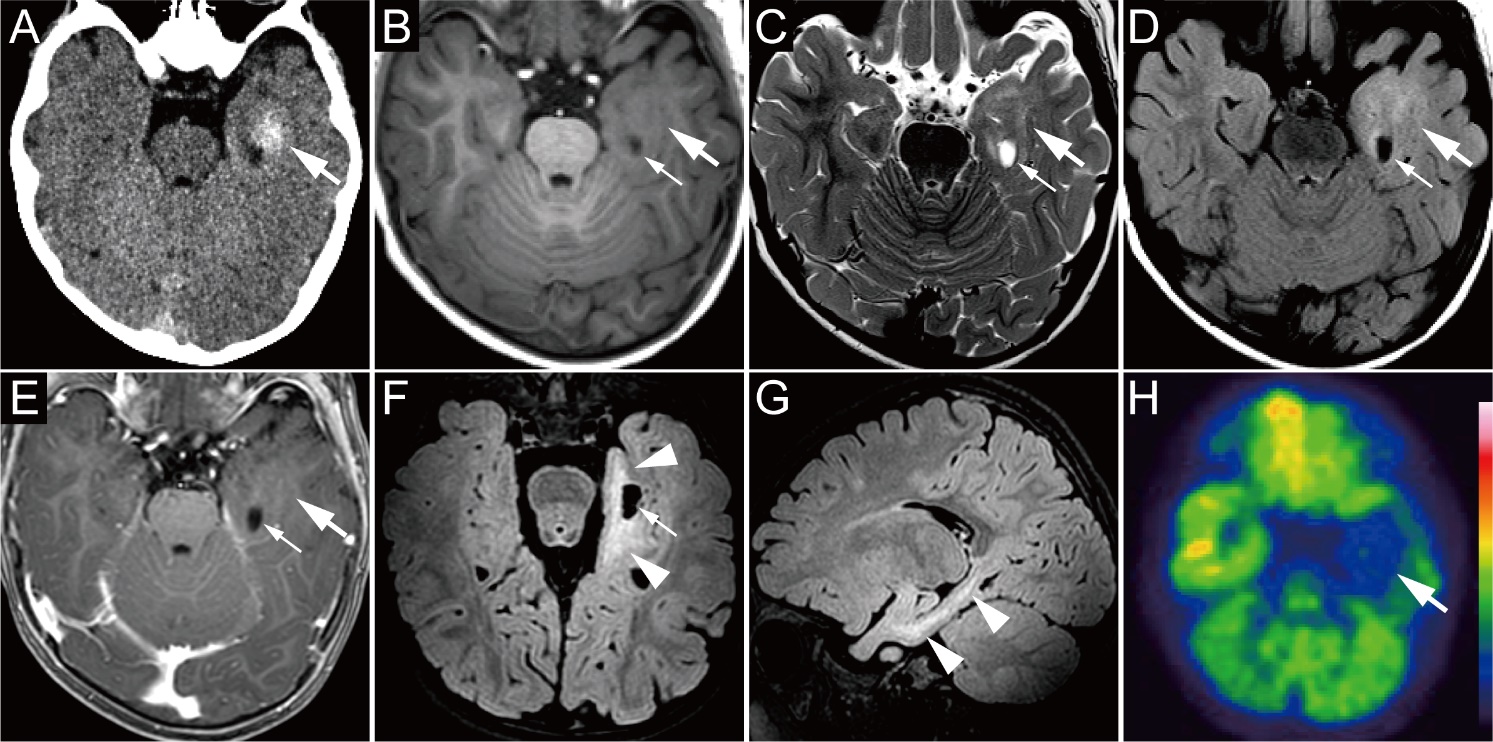


**Supplementary fig. 1 (A–H)** A 2-year-old girl with Group 1 *BRAF* V600E-mutant low-grade epilepsy-associated neuroepithelial tumor, pathologically diagnosed as pleomorphic neuroepithelial tumor of young (Patient 41). (**A)** CT scan shows calcifications in the left medial temporal lobe (*arrow*). (**B–G)** Axial T1-weighted (B), axial T2-weighted (C), axial FLAIR (D), axial gadolinium-enhanced T1-weighted (E), oblique FLAIR (F), and sagittal FLAIR images (G) demonstrate an ill-defined, band-shaped tumor with abnormal signals distributed along the medial temporal base (*arrowheads*). The tumor has iso-intensity on the T1-weighted image, slightly high intensity on the T2-weighted image, and high intensity on FLAIR images with no enhancement. Only the anterior part of the tumor has a mass effect, where calcifications were observed (*arrows*). The tumor contains flaccid cysts near the calcifications (*small arrows*). (**H)** Axial FDG-PET image demonstrates decreased uptake in the left medial temporal lobe (*arrow*). Color bar: standardized uptake values; top=9.21 and bottom=0.00. FDG, fluorodeoxyglucose; FLAIR, fluid-attenuated inversion recovery; PET, positron emission tomography


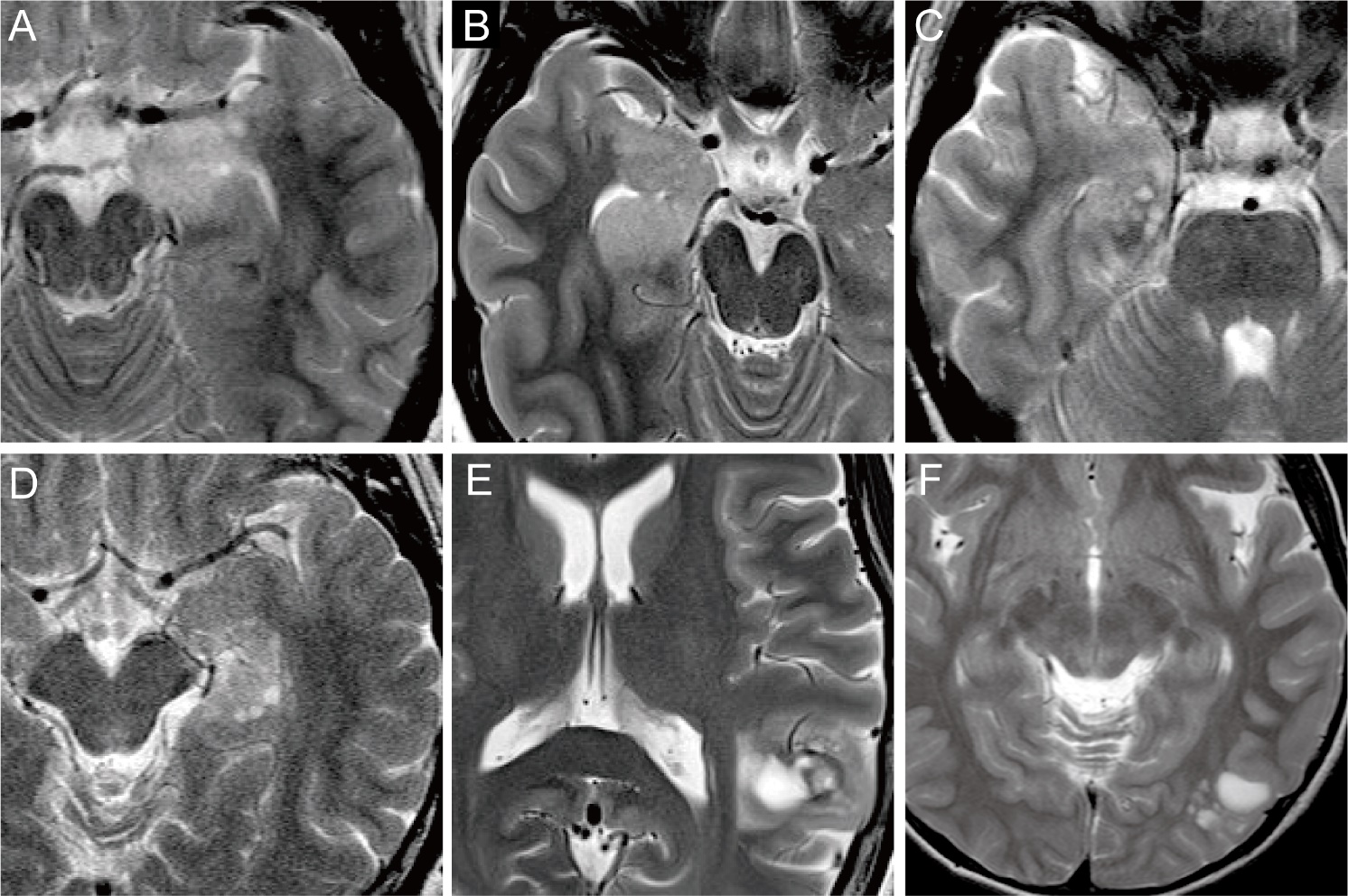


**Supplementary Fig. 2** T2-weighted magnetic resonance images of Group 3 low-grade epilepsy-associated neuroepithelial tumors with genotypes other than *BRAF* V600E mutation. **(A)** A 10-year-old boy with *BRAF* V504_R506 duplication (Patient 18). **(B)** A 15-year-old girl with *BRAF* T599dup (Patient 31). **(C)** A 23-year-old woman with *BRAF* c.1802_1810delAATCTCGAT insGTC (Patient 16). **(D)** A 34-year-old man with *KIAA1549*-*BRAF* fusion (Patient 5). **(E)** A 32-year-old woman with *BRAF* V600E and *CDKN2A/B* deletion (Patient 42). **(F)** A 5-year-old girl with *NF1* c3330_3333delTATG (Patient 28).

# Supplementary Table

**Supplementary Table S1. Neuroimaging findings of 46 patients with LEAT**

| Patient number | Neuroimaging group | MRI findings | | | | | | | | | | | | CT findings | | Other imaging modalities | |
| --- | --- | --- | --- | --- | --- | --- | --- | --- | --- | --- | --- | --- | --- | --- | --- | --- | --- |
|  |  | Location | Size (mm) | Borders | Shape | Exophytic growth | Mass effect^b^ | T1WI signal | T2WI signal | ADC map | Gadolinium enhancement^c^ | Cystic component | Hippocampal sclerosis | Skull scalloping | Calcifications | FDG-PET | ECD-SPECT |
| **1** | 1 | L, mT* | 31×18×17 | Indistinct | Band | - | - | Iso | Slightly high | NA | NA | + (Flaccid) | - | - | - | NA | NA |
| **2** | 1 | L, mT* | 39×22×20 | Indistinct | Band | - | - | Iso | Slightly high | NA | NA | + (Flaccid) | - | - | + | NA | NA |
| **3** | 1 | L, mT | 28×13×14 | Indistinct | Wedge | + | - | Iso | Slightly high | NA | NA | - | - | + | + | NA | NA |
| **4** | 2 | L, mT | 19×16×18 | Sharp | Round | - | ++ | Very low | Very high | NA | - | - | - | - | - | NA | NA |
| **5** | 3 | L, mT | 28×18×16 | Indistinct | Multilocular | - | ++ | Slightly low | Slightly high | NA | NA | + (Flaccid) | - | - | + | NA | NA |
| **6** | 1 | L, mT* | 56×22×24 | Indistinct | Band | - | + | Iso | Slightly high | Slightly high | - | + (Flaccid) | - | NA | NA | NA | NA |
| **7** | 1 | R, mT | 28×26×23 | Indistinct | Band | - | + | Iso | Slightly high | NA | NA | + (Flaccid) | NA | - | + | NA | NA |
| **8** | 1 | L, mT | 37×26×20 | Indistinct | Band | - | + | Iso | Slightly high | NA | NA | + (Flaccid) | - | - | + | NA | NA |
| **9** | 2 | L, F | 40×35×35 | Sharp | Round | + | ++ | Very low | Very high | NA | + | + (Tense) | - | - | + | NA | NA |
| **10** | 1 | R, mT | 45×23×18 | Indistinct | Band | - | + | iso | Slightly high | NA | NA | + (Flaccid) | - | - | + | NA | NA |
| **11** | 1 | L, lT | 21×14×13 | Indistinct | Wedge | - | - | Iso | Slightly high | NA | NA | + (Flaccid) | - | - | - | NA | ↓ |
| **12** | 1 | L, mT | 30×15×17 | Indistinct | Band | - | + | Iso | Slightly high | Slightly high | - | + (Flaccid) | - | - | + | NA | NA |
| **13** | 2 | L, F-I | 61×31×48 | Sharp | Rectangular | + | ++ | Very low | Very high | Very high | - | - | - | - | - | NA | ↓ |
| **14** | 3 | R, mT | 27×21×16 | Sharp | Round | - | ++ | Slightly low | High | NA | ++ | - | - | - | + | NA | NA |
| **15** | 1 | L, mT* | 23×23×20 | Indistinct | Band | - | - | Iso | Slightly high | NA | - | + (Flaccid) | - | - | + | NA | NA |
| **16** | 3 | R, mT | 26×16×11 | Indistinct | Multilocular | - | ++ | Slightly low | Slightly high | NA | - | - | - | - | - | NA | NA |
| **17** | 1 | L, lT | 18×16×8 | Indistinct | Band | - | - | Iso | Slightly high | NA | NA | + (Flaccid) | - | - | - | NA | NA |
| **18** | 3 | L, mT | 22×22×21 | Indistinct | Round | - | ++ | Slightly low | High | Slightly high | + | - | - | - | - | NA | ↓ |
| **19** | 1 | R, P | 34×16×18 | Indistinct | Wedge | - | - | Iso | High | NA | NA | + (Flaccid) | - | - | - | NA | NA |
| **20** | 3 | R, mT | 27×23×18 | Sharp | Round | - | ++ | Slightly low | Slightly high | NA | ++ | + (Tense) | - | - | + | NA | ↓ |
| **21** | 2 | L, mT | 70×33×41 | Sharp | Triangular | + | ++ | Slightly low | Very high | NA | - | + (Tense) | - | - | - | NA | NA |
| **22** | 2 | R, P-O | 43×41×27 | Sharp | Round | + | ++ | Very low | Very high | NA | NA | + (Tense) | - | - | - | ↓ | ↓ |
| **23** | 1 | R, mT* | 42×28×20 | Indistinct | Wedge | - | + | Iso | High | NA | NA | + (Flaccid) | + | - | + | ↓ | NA |
| **24** | 1 | R, lT* | 18×11×4 | Indistinct | Band | - | - | Iso | Slightly high | Slightly high | - | - | - | - | - | NA | → |
| **25** | 1 | L, mT* | 49×32×25 | Indistinct | Band | - | + | iso | Slightly high | Slightly high | - | + (Flaccid) | - | - | + | ↓ | ↓ |
| **26** | 1 | R, lT | 27×17×13 | Indistinct | Wedge | - | - | Iso | Slightly high | Slightly high | + | + (Flaccid) | - | - | - | ↓ | ↓ |
| **27** | 2 | R, F-I | 65×52×49 | Sharp | Round | + | ++ | Very low | Very high | Very high | - | - | - | + | + | ↓ | ↓ |
| **28** | 3 | L, T-O | 23×18×20 | Sharp | Multilocular | - | ++ | Slightly low | High | Slightly high | - | + (Tense) | - | - | + | ↓ | ↓ |
| **29** | 1 | R, lT | 18×17×13 | Indistinct | Wedge | - | - | Iso | Slightly high | Slightly high | NA | + (Flaccid) | - | - | + | ↓ | ↓ |
| **30** | 1 | L, mT | 14×11×6 | Indistinct | Band | - | - | Iso | Slightly high | Slightly high | - | + (Flaccid) | + | - | - | ↓ | ↓ |
| **31** | 3 | R, mT | 50×21×23 | Indistinct | Round | - | ++ | Slightly low | High | Slightly high | NA | - | - | - | - | ↓ | ↓ |
| **32** | 1 | R, lT* | 33×26×21 | Indistinct | Wedge | - | - | Iso | Slightly high | Slightly high | - | + (Flaccid) | - | - | + | ↓ | NA |
| **33** | 1 | R, mT* | 24×21×12 | Indistinct | Band | - | - | Iso | Slightly high | Slightly high | - | + (Flaccid) | - | - | + | ↓ | NA |
| **34** | 1 | R, lT | 23×12×6 | Indistinct | Wedge | - | - | Iso | Slightly high | Slightly high | - | + (Flaccid) | - | - | - | ↓ | NA |
| **35** | 1 | R, mT | 24×21×13 | Indistinct | Band | - | + | Iso | Slightly high | Slightly high | + | + (Flaccid) | - | - | + | ↓ | ↓ |
| **36** | 2 | R, P | 41×38×39 | Sharp | Round | + | ++ | Very low | Very high | Very high | - | - | - | + | - | ↓ | ↓ |
| **37** | 1 | L, lT | 28×20×18 | Indistinct | Wedge | - | - | Iso | Slightly high | Slightly high | NA | + (Flaccid) | - | - | - | ↓ | ↓ |
| **38** | 1 | L, mT* | 46×15×19 | Indistinct | Band | - | + | Iso | Slightly high | Slightly high | NA | + (Flaccid) | - | - | + | ↓ | NA |
| **39** | 1 | R, mT* | 27×14×23 | Indistinct | Band | - | + | Iso | Slightly high | Slightly high | - | + (Flaccid) | - | - | + | ↓ | ↓ |
| **40** | 1 | R, mT | 37×22×24 | Indistinct | Band | - | - | Iso | Slightly high | Slightly high | - | + (Flaccid) | - | - | - | ↓ | NA |
| **41** | 1 | L, mT* | 60×23×13 | Indistinct | Band | - | + | Iso | Slightly high | Slightly high | - | + (Flaccid) | + | - | + | ↓ | NA |
| **42** | 3 | L, T-O | 34×23×20 | Indistinct | Round | - | ++ | Slightly low | Slightly high | Slightly high | ++ | + (Tense) | - | - | - | ↑ | NA |
| **43** | 2 | R, F | 43×38×33 | Sharp | Round | + | ++ | Very low | Very high | Very high | - | - | - | + | - | ↓ | NA |
| **44** | 1 | L, mT | 25×18×18 | Indistinct | Wedge | - | + | Iso | Slightly high | Slightly high | + | - | - | - | + | ↓ | NA |
| **45** | 1 | R, lT* | 36×35×17 | Indistinct | Band | - | - | iso | High | Slightly high | - | + (Flaccid) | - | - | - | ↓ | NA |
| **46** | 1 | R, mT* | 52×28×27 | Indistinct | Band | - | + | iso | Slightly high | Slightly high | + | + (Flaccid) | - | - | + | ↓ | NA |

ADC, apparent diffusion coefficient; CT, computed tomography; ECD-SPECT, ethyl-cysteinate-dimer single photon emission computed tomography; F, frontal lobe; FDG-PET, fluorodeoxyglucose positron emission tomography; l, lateral; L, left; LEAT, low-grade epilepsy-associated neuroepithelial tumors; m, medial; MR, magnetic resonance; NA, not available; O, occipital lobe; P, parietal lobe; I, insular gyri; R, right; T, temporal lobe; T1WI, T1-weighted imaging; T2WI, T2-weighted imaging

^a^Distribution along the temporal base. ^b^Mass effect: -, no mass effect; +, partial mass effect; ++, diffuse mass effect. ^c^Gadolinium enhancement: -, no enhancement; +, faint and partial enhancement; ++, homogeneous enhancement.
